# Bio-Stimulating Effect of Natural Polysaccharides from *Lobularia maritima* on Durum Wheat Seedlings: Improved Plant Growth, Salt Stress Tolerance by Modulating Biochemical Responses and Ion Homeostasis

**DOI:** 10.3390/plants11151991

**Published:** 2022-07-30

**Authors:** Mohamed Taieb Bouteraa, Avinash Mishra, Walid Ben Romdhane, Anis Ben Hsouna, Kadambot H. M. Siddique, Rania Ben Saad

**Affiliations:** 1 Biotechnology and Plant Improvement Laboratory, Centre of Biotechnology of Sfax, University of Sfax, B.P ‘1177’, Sfax 3018, Tunisia; bouteraa.taieb@gmail.com (M.T.B.); walid.brm3@gmail.com (W.B.R.); benhsounanis@yahoo.fr (A.B.H.); 2 CSIR—Central Salt and Marine Chemicals Research Institute, Bhavnagar 364002, India; avinash@csmcri.res.in; 3 Plant Production Department, College of Food and Agricultural Sciences, King Saud University, P.O. Box 2460, Riyadh 11451, Saudi Arabia; 4 Departments of Life Sciences, Faculty of Sciences of Gafsa, Zarroug, Gafsa 2112, Tunisia; 5 The UWA Institute of Agriculture, UWA School of Agriculture and Environment, The University of Western Australia, Perth, WA 6001, Australia

**Keywords:** *Lobularia maritima*, polysaccharide, durum wheat, biostimulant, antioxidant enzymes, salt stress

## Abstract

Bioactivities of polysaccharides derived from halophyte plants have gained attention in recent years. The use of biostimulants in agriculture is an innovative method of dealing with environmental stressors affecting plant growth and development. Here, we investigated the use of natural polysaccharides derived from the halophyte plant *Lobularia maritima* (PSLm) as a biostimulant in durum wheat seedlings under salt stress. Treatment with polysaccharide extract (0.5, 1, and 2 mg/mL PSLm) stimulated in vitro wheat growth, including germination, shoot length, root length, and fresh weight. PSLm at 2 mg/mL provided tolerance to plants against NaCl stress with improved membrane stability and low electrolyte leakage, increased antioxidant activities (catalase (CAT), peroxidase (POD), and superoxide dismutase (SOD)), enhanced leaf chlorophyll fluorescence, proline, and total sugar contents, decreased lipid peroxidation (MDA), and reactive oxygen species (H_2_O_2_) levels, and coordinated the efflux and compartmentation of intracellular ions. The expression profile analyses of ten stress-related genes (NHX1, HKT1.4, SOS1, SOD, CAT, GA20-ox1, GA3-ox1, NRT1.1, NRT2.1, and GS) using RT-qPCR revealed the induction of several key genes in durum wheat growing in media supplemented with PSLm extract, even in unstressed conditions that could be related to the observed tolerance. This study revealed that PSLm extract contributes to salt tolerance in durum wheat seedlings, thereby enhancing their reactive oxygen species scavenging ability, and provided evidence for exploring PSLm as a plant biostimulant for sustainable and organic agriculture.

## 1. Introduction

The halophyte species, representing around 1% of the world’s flora, are highly salt-tolerant plants found in various saline biotopes, including coastal salt marshes [1,2,3]. Some 2500 halophyte species occur in saline coastal environments and land deserts worldwide and several have potential as cash crops (fuel, edible plants, fodder, medicine, chemicals, and ornamentals) [4,5]. Furthermore, the ability of halophytes to desalinate soil by absorbing and accumulating large amounts of salt enhances nutritional availability and produces favorable microclimates for glycophytes, contributing to ecosystem productivity [6]. Interestingly, an expanding number of pharmacological reports reveal that halophytic plants have a variety of beneficial impacts, including nutraceutical value [7,8,9], anti-fatigue [10], anti-oxidant [11,12], neuroprotective [13], anti-Alzheimer [14], anti-viral [15], and anti-inflammatory effects [16], partly attributed to several bioactive components. However, the severity of salt stress can disturb physiological and metabolic processes, decreasing crop growth and development. Sustainable strategies are needed to enhance plant tolerance against abiotic stresses. Plants respond and adapt to salt stress using a range of biochemical and developmental changes, including the synthesis of stress hormones and secondary metabolites, such as vitamins, terpenoids, flavonoids, phenolics, polysaccharides, saponins, fatty acids, proteins, glycosides, and trace elements, that prevent denaturation and oxidative damage [17]. These chemical compounds have anti-oxidant, anti-microbial, anti-inflammatory, and anti-tumoral benefits, and thus may play an important role in preventing a wide range of diseases [17]. Biostimulants are novel agronomic methods that have the potential to reduce cellular damage produced by abiotic stressors and to enhance crop tolerance [18]. Enzymes, humic substances, trace elements, carbohydrates, PGP-rhizobacteria, and seaweed extracts are the common forms of biostimulants [19,20]. Many agronomic studies have demonstrated the important role of algal formulations in protecting and improving various agricultural crops, inducing plant resistance to several biotic and abiotic stresses [20,21]. Carbohydrates extracted from halophytic plants have received recent attention from researchers in the developing polysaccharide field and are thought to contribute to the therapeutic benefits of halophytes [22]. Many types of bioactive polysaccharides have been extracted and purified from various halophyte plant parts using various extraction methods [23,24]. These polysaccharides represent a group of bio-macromolecules with a broad range of activities, such as hypoglycemic, hypolipidemic, cytoprotective, anti-tumor, immunity-modulatory, anti-oxidant, and hepato-protective activities [25,26,27].

In the face of climate change, rising world population, and soil salinity, research and development into producing stress-tolerant plants with high nutritional quality is becoming increasingly important. Halophyte plants, as vital constituents of saline farming and as a promising crop in their own right, are major sources of salt-resistance genes that could help researchers boost the tolerance of conventional crops to salinity. No studies have reported on the potential effects of polysaccharide extracts from halophyte *L. maritima* as a biostimulant and agent for promoting plant tolerance to abiotic stressors.

Consequently, this study evaluated the impact of PSLm on the development, salt tolerance, and anti-oxidant status of durum wheat seedlings subjected to salt stress (150 mM NaCl). Moreover, we aimed to contribute to the understanding of the regulatory mechanism of PSLm in the improvement of durum wheat salt stress resistance in terms of osmotic regulation, ion transport, and redox homeostasis. This study provides a sustainable, efficacious, and practical approach using PSLm extract to improve salinity tolerance in economically valuable crops.

## 2. Results

### 2.1. Chemical Analysis of Natural Polysaccharides from L. maritima

Table 1 summarizes the results of the chemical analyses for crude polysaccharide extract from *L. maritima*. In this study, PSLm revealed high uronic acid content (22%) and phenolic compounds (54.85 ± 2.56 mg/g gallic acid equivalents), and low total flavonoid content (2.33 ± 0.75 mg/g quercetin equivalents).

### 2.2. Effect of PSLm on Durum Wheat Seed Germination and Growth Rate

**Figure 1:**
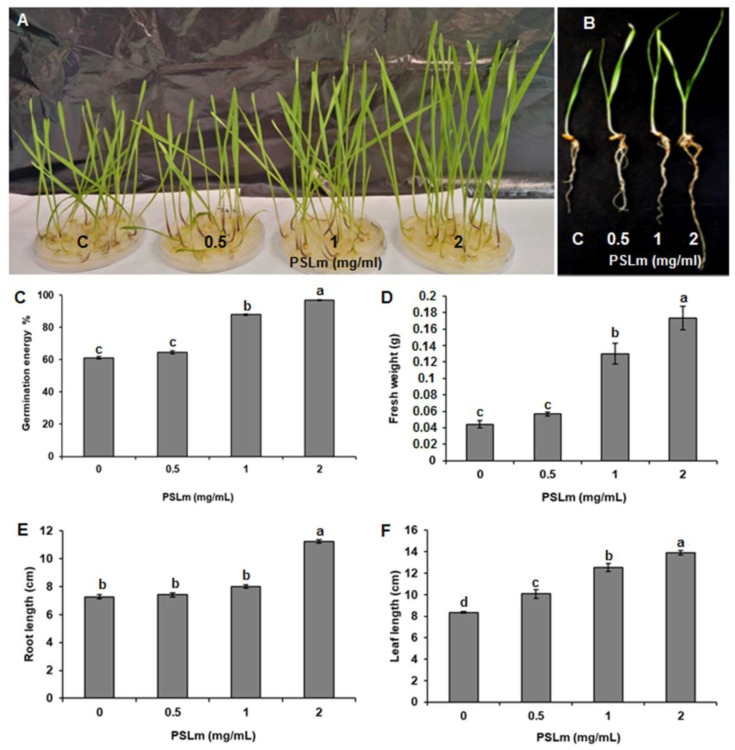
Polysaccharides derived from *L. maritima* (PSLm) enhanced seed germination and plant phenotypes of durum wheat cv. Karim. (**A**,**B**) Photographs taken eight days after sowing seeds treated with different PSLm concentrations (0.5, 1, and 2 mg/mL). Effect of increasing PSLm concentrations on (**C**) germination energy (GE) percentage, (**D**) fresh weight, (**E**) root length, and (**F**) leaf length. C—Control (untreated seedlings). Values are means ± SE of three biological replicates. Means sharing the same letter do not significantly differ at *p* < 0.05. Thirty seeds were assessed for each treatment replicate.

Three PSLm concentrations (0.5, 1, and 2 mg/mL) were tested to assess the effect of PSLm on durum wheat germination (Figure 1A,B). The 0.5 mg/mL PSLm treatment had no inhibitory effect on seed germination and behaved like the controls (Figure 1A,B). For the 1 and 2 mg/mL PSLm treatments, wheat germinated three days earlier than the control treatment (without PSLm) (with 64% and 96% germination, respectively) (Figure 1C). Interestingly, the 2 mg/mL PSLm treatment had the highest GE in the first three days of germination, suggesting a positive impact on durum wheat seed germination (Figure 1C). The 2 mg/mL PSLm treatment also increased leaf and root lengths and FW more than the 0.5 and 1 mg/mL PSLm treatments (Figure 1D–F), suggesting that 2 mg/mL PSLm is the optimum concentration for durum wheat seed germination and may act as a non-toxic biostimulant for plant growth.

### 2.3. Effect of Optimum PSLm Concentration on Wheat Seedlings under Salt Stress

#### 2.3.1. Plant Growth and Chlorophyll Fluorescence

**Figure 2:**
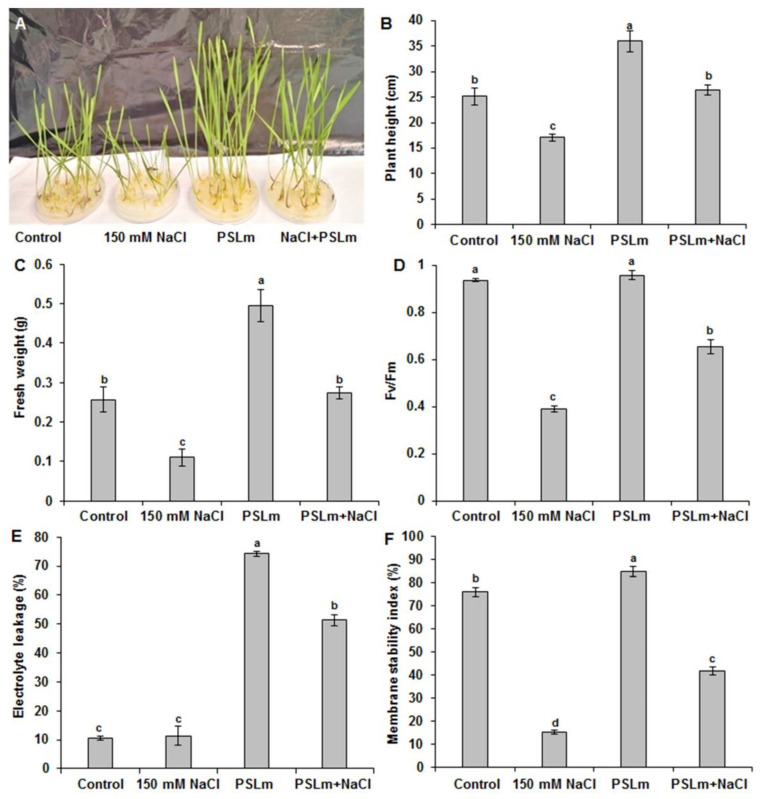
Effect of salt stress (150 mM NaCl) on plant phenotype (**A**), (**B**) plant height, (**C**) fresh weight, (**D**) chlorophyll fluorescence (Fv/Fm), (**E**) electrolyte leakage, and (**F**) membrane stability index of wheat seedlings in half-strength MS medium supplemented with (2 mg/mL) or without PSLm extract for 20 days. Values are means ± SE of three replicates. Different letters indicate significant differences at *p* < 0.05.

To examine the effect of 2 mg/mL PSLm on the salt tolerance of plants, four-day-old durum wheat plants were grown on half-strength MS plates containing 0 and 150 mM NaCl supplemented or not with 2 mg/mL PSLm. Twenty days later, the seedlings were captured and their physiological parameters were measured (Figure 2).

Under optimal conditions, no discernable difference in the growth was monitored in the durum wheat seedlings (Figure 2A). However, the seedlings grown in MS + 2 mg/mL PSLm exhibited a significant improvement in plant height and FW (Figure 2A–C). Interestingly, the seedlings subjected to salt stress exhibited decreased growth rate compared with their counterpart in normal conditions, while a discernably greater growth rate was monitored for the plants treated with PSLm in salinity conditions. In fact, as shown in Figure 2A, the addition of 2 mg/mL PSLm in MS medium was able to quench the effect of high salinity (150 mM). In addition, salt-stressed wheat seedlings supplemented with 2 mg/mL PSLm had higher chlorophyll fluorescence Fv/Fm (66%) than those not receiving PSLm (44%) (Figure 2D), which suggests that PSLm contributes to the maintenance of higher photochemical efficiency of photosystem II even under salinity.

#### 2.3.2. Osmolyte Accumulation

Salt-stressed wheat seedlings had significantly higher TSS and proline contents than unstressed seedlings (Figure S1). Salt-stressed wheat seedlings treated with 2 mg/mL PSLm had 57% and 63% higher TSS and proline contents, respectively, than those receiving no PSLm. Indeed, the PSLm treatments enhanced TSS and proline contents in the absence (2.8-fold and 3.75-fold) or presence (2-fold and 2.14-fold) of salt stress (Figure S1).

#### 2.3.3. Electrolyte Leakage and Membrane Stability Index

Electrolyte leakage (EL) (Figure 2E) and membrane stability index (MSI) (Figure 2F) did not significantly differ between wheat seedlings treated with 2 mg/mL PSLm and the control. However, salt-stressed wheat seedlings had significantly higher EL and lower MSI than unstressed seedlings (Figure 2F). Salt-stressed wheat seedlings treated with 2 mg/mL PSLm had higher MSI and lower EL than those receiving no PSLm (Figure 2F).

#### 2.3.4. Lipid Peroxidation and Antioxidant Enzyme Activities

**Figure 3:**
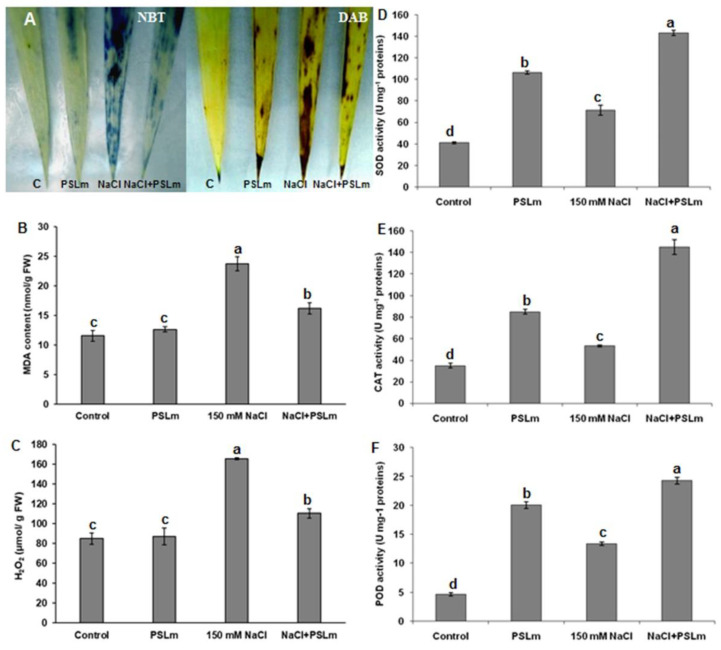
O_2_^−^ localization by NBT staining (upregulated) and H_2_O_2_ localization by DAB staining (downregulated) (**A**), (**B**) MDA content, (**C**) H_2_O_2_ content, (**D**) SOD activity, (**E**) CAT activity, and (**F**) POD activity in control and salt-stressed (150 mM NaCl) durum wheat seedlings cultured in half-strength MS medium supplemented with (2 mg/mL) or without PSLm extract for 20 days. Values are means ± SEM (n = 3). Means sharing the same letter do not significantly differ at *p* < 0.05.

Under normal conditions (MS), control seedlings showed weak NBT and DAB staining, while those treated with 2 mg/mL PSLm showed moderate staining, indicating that PSLm application may accumulate superoxide to some extent. Interestingly, salt-stressed seedlings with no PSLm had more intense NBT and DAB coloration in leaves than salt-stressed seedlings treated with 2 mg/mL PSLm (Figure 3A). Thus, salt stress significantly increased MDA (Figure 3B) and H_2_O_2_ (Figure 3C) contents in wheat seedling leaves; an exogenous application of 2 mg/mL PSLm to salt-stressed seedlings reduced MDA and H_2_O_2_ contents by 40% and 37%, respectively, compared with those grown under standard conditions (Figure 3B,C).

To mitigate the harmful effects of salt-induced oxidative stress, plants can activate antioxidative defense including enzymatic or/and non-enzymatic systems. Indeed, in the case of the plants grown in the MS medium supplemented with 2 mg/mL, there was an increase in the SOD (Figure 3D), CAT (Figure 3E), and POD (Figure 3F) activities even under unstressed conditions and these activities remained significantly higher than in the control plants. Salt stress alone increased SOD, CAT, and POD enzymes activities 1.5-fold, 1.3-fold, and 2.8-fold, respectively, compared with unstressed plants. The corresponding increases for salt-stressed seedlings supplemented with 2 mg/mL PSLm were 3.5-fold, 3.68-fold, and 4.8-fold, in CAT, SOD, and POD activities, respectively, compared with those grown under normal conditions (MS only) (Figure 3D–F).

#### 2.3.5. Na^+^ and K^+^ Accumulation in Different Tissues of Wheat Seedlings

**Figure 4:**
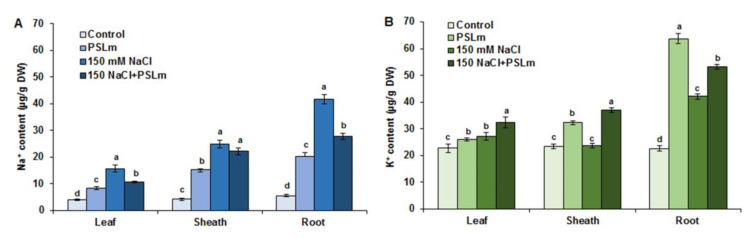
Na^+^ content (**A**) and K^+^ content (**B**) in leaves, sheaths, and roots under normal and salt-stressed (150 mM NaCl) durum wheat seedlings cultured in half-strength MS medium supplemented with (2 mg/mL) or without PSLm extract for 20 days. Values are means ± SEM (n = 3). Different letters indicate significant differences at *p* < 0.05.

Salt stress significantly increased Na^+^ contents in roots, sheaths, and leaves 8.4-fold, 5.2-fold, and 2.25-fold, respectively, compared with unstressed plants (Figure 4A) and K^+^ content in roots 1.8-fold, with no significant differences in sheaths and leaves (Figure 4B). PSLm-treated wheat seedlings subjected to salinity had lower tissue Na^+^ contents than those receiving no PSLm, but the values remained higher than unstressed seedlings. PSLm-treated wheat seedlings subjected to salinity displayed 2.95-fold and 1.36-fold higher root and sheath K^+^ contents, respectively, than unstressed seedlings (Figure 4B). Salt-stressed wheat seedlings treated with PSLm had 37%, 43%, and 75% lower Na^+^ contents in roots, sheaths, and leaves, respectively, than those receiving no PSLm (Figure 4A). In contrast, salt stress plus PSLm application increased K^+^ contents by 20%, 37%, and 15% in roots, sheaths, and leaves, respectively, compared with salt-stressed alone (Figure 4B).

#### 2.3.6. Expression Levels of Stress-Responsive Durum Wheat Genes

**Figure 5:**
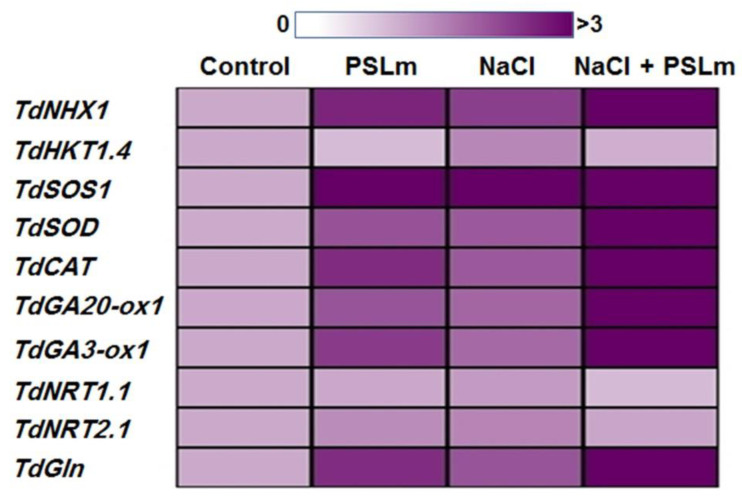
Heatmap depicting the expression pattern of ten stress-related genes (*NHX1*, *HKT1.4*, *SOS1*, *SOD*, *CAT*, *GA20-ox1*, *GA3-ox1*, *NRT1.1*, *NRT2.1*, and *GS*) under control and salt-stressed (150 mM NaCl) durum wheat seedlings cultured in 1/2 MS medium supplemented with (2 mg/mL) or without PSLm extract for 20 days. The data represent means of three independent experiments. Color code is presented above the heatmap.

To further insights into the molecular mechanisms of enhanced salt stress tolerance afforded by exogenous application of 2 mg/mL PSLm, real-time RT-qPCR analysis was performed on ten stress-related genes (NHX1, HKT1.4, SOS1, SOD, CAT, GA20-ox1, GA3-ox1, NRT1.1, NRT2.1, and GS) in leaves of durum wheat seedlings grown under normal and stress conditions. The real-time qPCR analysis revealed that salt-stressed wheat seedlings treated with PSLm had considerably higher transcript abundances of eight stress-related genes (NHX1, HKT1.4, SOS1, SOD, CAT, GA20-ox1, GA3-ox1, and GS) than both the controls and the stressed seedlings (Figure 5). In contrast, the PSLm treatments decreased the TdHKT1.4 gene expression, less so when combined with salt stress (Figure 5). Interestingly, the NRT2 gene expression did not significantly differ between stressed and unstressed seedlings (Figure 5).

## 3. Discussion

While synthetic chemical fertilizers play an important role and critical function in increasing crop yields and preventing plant diseases, they can adversely affect agriculture, the environment, and consumer health. Biostimulant products offer an attractive and promising solution to these adverse effects. Many biostimulants, such as plant growth-promoting bacteria, humic acids, silicon, chitosan, and seaweed extracts, are well established for plant protection and growth improvement [19]. Furthermore, biostimulants that induce tolerance to salt stress in crops could act by increasing plant water absorption capacity and/or stimulating the accumulation of substances with protective roles for cell membranes [18]. However, the mechanisms activated by biostimulants in response to stress conditions that limit plant growth and development by affecting physiological and biochemical processes are not well studied. Our research highlights the potential role of an exogenous supply of natural polysaccharide extract from the halophyte plant *L. maritima* (PSLm) to alleviate the adverse effects of salt stress on the growth of wheat seedlings, through improving antioxidant activities and regulating the efflux and compartmentation of intracellular ions.

We demonstrated that the addition of 1 and 2 mg/mL PSLm to the culture medium has no detrimental effect on seed germination and significantly enhanced GE and biomass components. Other studies have shown that polysaccharides from seaweed extracts improved growth and development in crops such as wheat [28], maize [29], rice [30], chickpea [31], and tomato [32,33]. In addition, all of them demonstrated plant improvement due to the high concentration of minerals, amino acids, polyphenols, and other components in aqueous seaweed extracts. Salt stress significantly hinders wheat seedling growth and development by decreasing root length, fresh and dry weights, and chlorophyll content [34]. We showed that 2 mg/mL PSLm is sufficient for enhancing tolerance to salt stress in durum wheat plants; however, its mechanism is complex and requires further investigation.

Proteins, sugars, and other macromolecules easily degrade under salt stress, damaging cell membranes and affecting osmotic pressure. PSLm-treated wheat seedlings had increased soluble sugar contents, suggesting that soluble sugars help maintain osmotic balance and stabilize cell membranes. Similarly, salt-stressed wheat seedlings had significantly higher proline contents than unstressed seedlings to suppress the osmotic imbalance generated by salinity. The PSLm application further increased the proline content in salt-stressed wheat seedlings to mitigate osmotic stress resulting from salinity. Biostimulants can increase the concentration of proline, simple sugars, alcohols, abscisic acid, and antioxidant compounds to decrease the damage caused by free radical accumulation [35]. Abiotic stresses can enhance biostimulant-activated metabolic pathways, allowing plants to adapt to or overcome stressful conditions [18]. Interestingly, enzymatic antioxidants are often activated in plants treated with biostimulants; these protective molecules are well recognized for decreasing the degenerative effects of free radicals that accumulate in plant tissues under abiotic stress [36,37].

Under salt-stress conditions, stressed plants exhibit a decrease in their photosynthetic efficiency and its chlorophyll fluorescence traits. Based on our findings, PSLm contributes to the alleviation of salt-stress negative effects on photosynthetic efficiency by increasing the chlorophyll fluorescence Fv/Fm value. It is possible that maintaining photosynthetic efficiency underlies the significant increases in soluble sugar contents in PSLm-treated wheat seedlings when subjected to salt stress.

Salt-stressed durum wheat seedlings treated with 2 mg/mL PSLm had significantly higher MSI and lower EL than those receiving no PSLm, suggesting that exogenous PSLm mitigates NaCl-induced oxidative damage. In other studies, polysaccharide extracts from *P. yezoensis* scavenged free radicals and prevented salt-stress-related lipid peroxidation caused by active oxygen [38,39]. Salt stress activates ROS (O_2_^−^, OH^•^, and H_2_O_2_) accumulation, leading to lipid peroxidation of chloroplast membranes and degradation of chlorophyll concentration [40]. The present study showed that salt stress increased MDA and H_2_O_2_ contents of wheat seedlings and decreased chlorophyll fluorescence (Fv/Fm), confirming that ROS caused damage to membrane lipids. However, salt-stressed wheat seedlings treated with PSLm had lower leaf MDA and H_2_O_2_ levels than those receiving no PSLm, indicating an overall reduction in oxidative stress. Consequently, we can hypothesize that PSLm helps plants mitigate the harmful effect of salt stress by stimulating ROS scavenging systems. In addition, PSLm contributes to the maintenance of the photosynthetic capacity at appropriate levels in salt-stressed plants, improving growth. Khan et al. [21] showed that seaweed extracts enhanced chlorophyll levels by increasing chlorophyll synthesis. Our results showed that an exogenous PSLm application decreased lipid peroxidation and prevented chlorophyll degradation in salt-stressed wheat seedlings.

Numerous studies have shown that SOD, POD, and CAT are closely associated with salt tolerance in plants [16,41,42,43]. In wheat, studies have reported that wheat plants increase antioxidant enzyme activities (e.g., SOD, CAT, APX, POX, and GR) under various abiotic stresses to protect against oxidative damage [44,45]. Similarly, we found significantly higher SOD, CAT, and POD activities in salt-stressed plants than unstressed plants. The PSLm-treated plants with or without NaCl stress increased SOD, POD, and CAT activities relative to no PSLm, protecting the wheat seedlings from oxidative stress. These results confirm that PSLm enhances ROS scavenging in wheat seedlings by regulating antioxidant enzyme activities and inducing defense responses under salt stress. Interestingly, the exogenous PSLm application also increased proline content. Thus, pre-treatment with PSLm induces proline and modulates enzyme activities, reducing ROS directly and protecting plants from salt stress, as reported by Zou et al. [28,39].

The ability to restrict leaf Na^+^ transport and accumulation is the most important plant adaptation to salt stress [46]. Wheat is a classic ‘salt excluder’, characterized by low rates of Na^+^ transport to leaves, thus keeping mesophyll cells relatively Na^+^-free [47,48,49]. In this study, roots accumulated large amounts of Na^+^ relative to other tissues under salt stress. However, exogenous PSLm application significantly decreased leaf Na^+^ content relative to the salt-stressed plants receiving no PSLm. Thus, PSLm-treated wheat seedlings subjected or not to salt stress excluded Na^+^ selectively from leaves, the primary photosynthetic tissue [50]. Under salt stress, plants suffer from K+ imbalance due to the competitive inhibition of its uptake by Na^+^, coupled with high Na^+^/K^+^ ratios that disrupt cellular homeostasis [46].

In addition, high K^+^ selectivity plays a crucial role in salt tolerance in wheat. Plants require potassium to maintain membrane potential and swelling pressure, activate enzymes, and regulate osmotic pressure, stomatal movement, and orientation. [51]. In this study, PSLm-treated plants with or without salt stress had high root K^+^ contents, contributing to the salt tolerance.

Several types of transporters and/or channels mediate transmembrane transport of Na^+^ and K^+^ in plants [52], some of which are linked to leaf Na^+^ exclusion, including high-affinity K^+^ transporters (HKTs). In bread wheat, TaHKT2;1 is thought to play a role in Na^+^ absorption from the soil [53]. Kumar et al. [54] showed that the TaHKT2;1 transcript level was upregulated in the shoots of the salt-sensitive genotype but downregulated in the salt-tolerant genotype. In our study, salt stress upregulated the TdHKT1;4 gene expression, while PSLm significantly downregulated its expression in salt-stressed wheat seedlings. However, wheat seedlings treated with PSLm with or without salt stress significantly upregulated TdSOS1 and TdNHX1 gene expressions. These results are similar to those of Zou et al. [28], who reported significantly higher TaSOS1 and TaNHX2 transcripts in salt-stressed plants treated with LNP-2 than those not treated with LNP-2, thus alleviating salt stress damage. Indeed, the upregulation of CAT and SOD genes in seedlings exposed to PSLm or salt stress could increase the capacity of plants to alleviate ROS. GA20- and GA3-oxidases are key enzymes in the pathway generating bioactive GAs, while GA2-oxidase is critical for maintaining GA homeostasis [55,56]. We found that salt-stressed plants treated with PSLm had an upregulated expression of GA metabolism-related genes (GA20ox and GA3ox) relative to those receiving no PSLm, suggesting that PSLm is involved in the upregulation of the GA metabolism-related genes (GA20ox and GA3ox) to regulate GA homeostasis under salt stress.

Wang et al. [57] showed that changes in glutamine synthetase (GS) activity and gene expression (TaGS1 and TaGS2) underlie the effect(s) of osmotic stress on nitrogen metabolism, which could explain the biomass reduction. During water deficit, wheat significantly decreased GS2 abundance and activity in its youngest leaves [58]; GS could be a good metabolic indicator of drought stress tolerance in wheat. Indeed, drought-tolerant wheat genotypes had higher GS activity and transcript abundance of both GS isoforms than susceptible varieties [59]. Similarly, drought stress decreased GS activity in drought-susceptible wheat cultivars [60]. Our results confirmed that salt stress upregulated the TdGS expression in durum wheat cv. Karim.

Although PSLm is rich in active compounds, the precise biomolecule(s) responsible for ameliorating the stress response has not been elucidated. Recently, Zou et al. [28] reported that polysaccharides extracted from brown algae (*Lessoniani grescens*) induced antioxidant enzyme activities to protect salt-stressed wheat, related mainly to uronic acids and sulfates. Polyphenols, another major compound in PSLm extract, have high antioxidant capacities and metal-chelating activities. The biostimulants benefits are related to organic molecules, phytohormones, amino acids, phenolic compounds, and bioactive secondary metabolites [61]. The richness of bioactive compounds—carbohydrates (85%) and polyphenols (54.85 mg/g GAE)—in PSLm offers an alternative solution for mitigating salt stress, likely due to stimulating the mechanisms involved in salt tolerance, such as ROS scavenging systems.

## 4. Materials and Methods

### 4.1. Chemical Analysis of Natural Polysaccharides

PSLm was previously extracted from the aerial parts of *L. maritima*, presenting high percentage of carbohydrates (~85%) and the estimated average molecular weight was 130.62 kDa [62]. Total uronic acids were estimated by m-hydroxydiphenyl (MHDP) assay [63] using D-glucuronic acid as standards. The sulfate content was quantified using the barium chloride BaCl_2_ method (standard: K_2_SO_4_) [64]. Total phenolic content was determined using Folin–Ciocalteu’s method [65]. Flavonoid contents were measured as per the Dowd method [66]. All determinations were performed in triplicate.

### 4.2. Plant Material, Growth Conditions, and Germination Assay

Seeds of durum wheat (*Triticum turgidum* L. var. *durum*) cultivar Karim were supplied by the Centre d’Appui Chebika-CRDA Kairouan in Tunisia. Cultivar Karim is one of the most used and productive durum wheat commercial genotypes under favorable environments [67]. Seeds were surface sterilized as described by Ben Saad et al. [68]. Thirty seeds were placed in triplicates on petri dishes with filter paper containing a half-strength MS medium supplemented with PSLm (0, 0.5, 1, or 2 mg/mL) to investigate the effect of PSLm on durum wheat germination. The petri dishes were transferred to a growth incubator set at a 14 h light/10 h dark photoperiod, 25 °C/20 °C day/night cycle, and 65% relative humidity. Germination energy (GE) was calculated as follows:

GE = (number of germinated seeds at day 3/total number of seeds) × 100(1)

After 10 days of growth, the seedlings were photographed and root, leaf lengths, and fresh weight (FW) were determined. The assays were conducted in triplicate using independent seed lots.

### 4.3. Effect of PSLm on Salt-Stressed Durum Wheat Seedlings

Four-day-old durum wheat seedlings were transferred to petri dishes containing the half-strength MS medium supplemented or not with 150 mM and containing either 0 or 2 mg/mL PSLm. After 20 days of salt stress, one sample was randomly collected from each group to determine plant height, fresh weight (FW), and osmolyte accumulation (total soluble sugar and proline contents), leaf chlorophyll fluorescence, oxidative stress markers, and transcript accumulation. Each group had three petri plates with 30 plants in each. Nutrient solutions were renewed daily.

### 4.4. Leaf Chlorophyll Fluorescence

Chlorophyll fluorescence (Fv/Fm) was measured in random fully expanded leaves that had been dark-adapted for 30 min before measurement with the portable photosynthesis system (Handy-Plant Efficiency Analyser, Hansatech Instruments) as described by Ben Saad et al. [69]. Measurements were performed on three visually healthy leaves per treatment.

### 4.5. Proline Content

Free proline concentration was determined in leaves using the method described by Ben Saad et al. [3]. Fresh leaves (0.1 g) were homogenized in 3% (*w*/*v*) sulfosalicylic acid. The homogenate was centrifuged at 10,000× *g* for 10 min at 4 °C, then 0.5 mL of supernatant was added to 0.5 mL of acidic ninhydrin reagent and 0.5 mL of ice-cold acetic acid. The mixture was incubated at 100 °C for 60 min. After incubation, 2 mL of toluene was added and mixed by shaking for extraction. The chromophore containing the toluene was aspirated and cooled to room temperature. Proline content in the supernatant was quantified spectrophotometrically at 520 nm, with the concentration calculated from a standard curve of pure proline. Values are expressed in µmol g^−1^ fresh weight (FW).

### 4.6. Total Soluble Sugar Content

Total soluble sugar (TSS) content was quantified in leaves using the anthrone method, as described by Ben Hsouna et al. [16]. Fresh tissue (0.2 g) was homogenized in ice-cold 80% ethanol and centrifuged at 10,000× *g* for 10 min at 4 °C. Then, 1 mL of supernatant was reacted with 3 mL of freshly prepared anthrone reagent by incubating the reaction mixture for 10 min at 100 °C in a hot water bath. The reaction was terminated by quick cooling in an ice bath, then allowed to cool at room temperature. Optical density was measured at 620 nm, with TSS content calculated from a standard curve prepared using D-glucose and expressed in µg g^−1^ FW.

### 4.7. H_2_O_2_ and MDA Contents and In Situ Histochemical Staining for O_2_^−^ and H_2_O_2_

Fresh leaves of 20-day-old durum wheat seedlings were harvested to measure MDA and H_2_O_2_ contents as described by Ben Saad et al. [70]. To measure MDA contents, fresh leaves were harvested, ground in 5 mL of 0.1% trichloracetic acid, and mixed with 5 mL of 0.5% thiobarbituric acid. Samples were then boiled for 10 min, cooled down to room temperature, and centrifuged at 1200× *g*. The cleared supernatant was analyzed by monitoring the difference in absorbance at 532 nm and 600 nm. To measure H_2_O_2_ levels, fresh tissues were homogenized with 0.5% (*w*/*v*) TCA and then centrifuged at 14,000× *g* for 15 min. An aliquot of the supernatant was added to a 100 mM phosphate buffer (pH 7.0) and 1 M KI. The reaction mixture was incubated in the dark for 1 h and the absorbance was determined at 390 nm.

In addition, fresh leaves of durum wheat were harvested to measure the accumulation of superoxide radicals (O_2_^−^) and hydrogen peroxide (H_2_O_2_) using NBT and DAB staining as described by Ben Saad et al. [71].

For H_2_O_2_ detection, leaves were treated with 1 mg/mL of 3, 30-diaminobenzidine (DAB) HCl, pH 3.8 (the pH was adjusted to 7.5 after solubilization) under vacuum infiltration for 5 min. After a dark incubation for 4 h under stirring, leaves were cleared by boiling the leaves in 80% (*v*/*v*) ethanol for 2 h and embedded in 10% (*v*/*v*) glycerol. The reaction of DAB with H_2_O_2_ resulted in a brown polymerization product, and the level of H_2_O_2_ accumulation correlated with the intensity of brownish stains in the leaves.

For O_2_^−^ detection, leaves were immersed in 1 mg/mL fresh NBT solution (prepared in 25 mM HEPES, pH 7.6) and subjected to vacuum infiltration for 5 min. Then, leaves were incubated under dark conditions for 2 h followed by a treatment with ethanol: acetic: acid: glycerol solution (3:1:1).

Each test was repeated three times using the leaves from three different plants in each treatment.

### 4.8. CAT, SOD, and POD Activities

Fresh leaves of 20-day-old durum wheat seedlings were harvested to determine CAT, SOD, and POD enzyme activities. A total of 200 mg of fresh leaves were homogenized at 4 °C with a 2 mL extraction buffer (0.1 M phosphate buffer, pH 7.5) for protein extraction and enzymatic assays. Soluble protein was assessed as described by Bradford [72].

Total CAT activity was measured spectrophotometrically according to the method of Aebi [73], by monitoring the decline in absorbance at 240 nm as H_2_O_2_ was consumed. The reaction mixture contained a 50 mM phosphate buffer (pH 7), to which 30 mM H_2_O_2_ was added. The reaction was initiated by adding an appropriate dilution of enzyme extract to this solution. One unit of CAT was defined as 1 µmol/mL H_2_O_2_ decomposed per minute.

Total SOD activity was assayed according to Harbaoui et al. [74]. An aliquot of crude enzyme extract was added to the reaction mixture at a final volume of 3 mL. The reaction mixture contained 50 mM K phosphate buffer (pH 7.8), 0.1 mM EDTA, 13 mM L-methionine, 2 mM riboflavin, and 75 mM NBT. The reaction was started by exposing the mixture to cool white fluorescent light for 15 min. One unit of SOD activity corresponds to the amount of enzyme, which causes 50% inhibition of the reduction in NBT as monitored at 560 nm.

Total POD activity was determined according to Maehly and Chance [75] by the guaiacol oxidation method. The reaction mixture contained 50 mM phosphate buffer (pH 7), 20 mM guaiacol, 40 mM H_2_O_2,_ and the protein extract. The increase in absorbance at 470 nm due to the guaiacol oxidation was recorded every 20 s. One enzyme unit of POD is defined as change in one unit of absorbance/min. Three biological replicates were measured and three independent experiments were analyzed.

### 4.9. Na^+^ and K^+^ Concentrations

Na^+^ and K^+^ accumulations were estimated in leaves, roots, and sheaths of durum wheat as described previously by Ben Romdhane et al. [76,77]. Healthy collected tissue was washed thoroughly in distilled water, dried at 80 °C for 72 h, and ground to a fine powder. For each sample, 50 mg leaf powder was incubated for 1 week in 0.5% HNO_3_ solution. Ion content was analyzed using an atomic absorption spectrometer (Thermo Scientific), with concentrations expressed as µg g^−1^ DW.

### 4.10. Expression Analysis of Genes Encoding Stress-Related Proteins and Na^+^/K^+^ Transporter

Total RNA was extracted from durum wheat seedlings using the RNAeasy Plant mini kit (Qiagen) as per the manufacturer’s protocol. Five micrograms (µg) of total RNA per sample were used to synthesize first-strand cDNA using SuperScriptTM III reverse transcriptase (Invitrogen), oligo-(dT18), and random hexamer primers according to the manufacturers’ instructions. Total cDNA from durum wheat was used in real-time qPCR reactions to analyze transcript accumulation of ten stress-related genes (NHX1, HKT1.4, SOS1, SOD, CAT, GA20-ox1, GA3-ox1, NRT1.1, NRT2.1, and GS) following the methods described by Ben Romdhane et al. [78]. Relative expression was determined using the (2−ΔΔCT) method [79], with the cell division control protein (AAA-superfamily of ATPases) (CDC gene) used as a housekeeping gene. Supplementary Table S1 lists the primers used. Three biological repetitions were performed to calculate the relative expression level.

### 4.11. Statistical Analyses

All data are provided as the mean ± standard error of means (SEM) of three independent replicates. Analysis of variance was performed using XLSTAT software with one-way ANOVA. According to Bonferroni’s post hoc test, the mean values marked with different letters on the figures significantly differ at *p* < 0.05.

## 5. Conclusions

Our results showed that natural polysaccharide extracts from *L. maritima* (PSLm) affect durum wheat seedlings’ salt stress response positively. Under salt stress, decreased ROS and lipid peroxidation, as well as enhanced proline content and antioxidant levels, may help to maintain higher photosynthetic efficiency and promote shoot and root growth. The findings highlight the importance of PSLm as a non-toxic solution for crop management. Furthermore, the expression level of several stress-related genes indicates that PSLm could regulate some stress-related genes, indirectly leading to the observed salt tolerance. This study revealed the possible role of PSLm in biochemical and molecular mechanisms that improve salt tolerance of durum wheat and contribute positively to crop cultivation and promotion by using halophytes resources.

## Figures and Tables

**Table 1:** Physicochemical characteristics of the crude polysaccharide extract of *L. maritima*.

Parameters	PSLm
Yield (%)	8.5 ± 0.89
Molecular weight (kDa)	130.62
PH	7.04 ± 0.025
Uronic acids (% DW)	22.3 ± 1.4
Total sugars (% DW)	84.85 ± 0.56
Sulfate (% DW)	35.5 ± 3.33
Phenolic compounds (mg/g gallic acid equivalents) Total flavonoids (mg/g quercetin equivalents)	54.85 ± 2.56 2.33 ± 0.75

Values are scored as means ± standard deviation of triplets.

## Data Availability

All data are contained within the article or supplementary materials.

## Supplementary Materials

The following are available online at https://www.mdpi.com/article/10.3390/plants11151991/s1, Figure S1. Effect of salt stress (150 mM NaCl) on total soluble sugars content (A) and proline content (B) of wheat seedlings in half-strength MS medium supplemented with (2 mg/mL) or without PSLm extract. Values are means ± SE of three replicates. Different letters indicate significant differences at *p* < 0.05; Table S1. Sequences of primers used for real-time analysis.
