# Supplementary material for: Bio-Stimulating Effect of Natural Polysaccharides from Lobularia maritima on Durum Wheat Seedlings: Improved Plant Growth, Salt Stress Tolerance by Modulating Biochemical Responses and Ion Homeostasis
Source: Plants (Basel). 2022 Jul 30;11(15):1991. doi: 10.3390/plants11151991 (PMC9370194; doi:10.3390/plants11151991)
Supplement: Supplementary file 1 [file plants-11-01991-s001.zip › plants-1763982-supplementary.pdf]

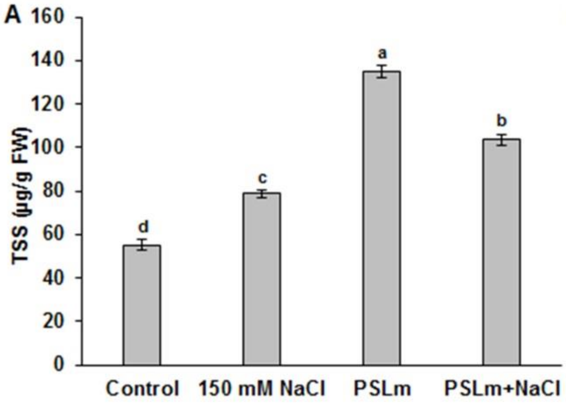

Figure S1. Effect of salt stress (150 mM NaCl) on total soluble sugars content (A) and proline content (B) of wheat seedlings in half-strength MS medium supplemented with (2 mg/mL) or without PSLm extract. Values are means ± SE of three replicates. Different letters indicate significant differences at $P < 0.05$.

Table S1. Sequences of primers used for real-time analysis.

Primers		Sequences	Accession no.
qRT-NHX1-F	*Sodium/hydrogen exchanger 1*	5'-GCAGCTGATATCTCAAGCCA-3'	AY296910.1
qRT-NHX1-R		5'- ACTGAAGGTTTGTTTGGGGG-3'	
qRT-HKT1,4-F	*High-affinity potassium transporters*	5′- GATGCTAGCGGCAACGGT-3′	KF443079.1
qRT-HKT1,4-R		5′- CGCGTTCGACACCTTCTC-3′	
qRT-SOS1-F	*Salt overly sensitive 1*	5′- AGAGTTTCCGGAGATCCAGC-3′	EU552490.1
qRT-SOS1-R		5′- TGCTGCCATACATGCTGACT-3′	
qRT-CAT-F	*Catalase*	5′-CGAGAAGATGGTGATCGAGA-3′	KP696753.1
qRT-CAT-R		5′-TGTTGATGAATCGCTCTTGC-3′	
qRT-SOD-F	*Superoxide dismutase*	5'-GCCATTGATGAGGATTTTGG-3'	KP696754.1
qRT-SOD-R		5'-CAAAGCTAGCCACACCCATC-3'	
qCDC-F	*Cell division control protein*	5′-GCCTGGTAGTCGCAGGAGGAT-3′	Ta54227
qCDC-R		5′-ATGTCTGGCCTGTTGGTAGC-3′	
qGA20ox1-F	*Gibberellin20 oxidase 1*	5′-CGCCTACCCGGACTTCAC-3′	XM_037588783
qGA20ox1-R		5′-TAAGTAAGTCATGTCCTGGCGG-3′	
qGA3ox1-F	*Gibberellin3-beta-dioxygenase 1*	5′- TTCTACAACCCGGCGTTCG-3′	XM_037599618
qGA3ox1-R		5′- TGCCTTTGGCGCCTTGAC-3′	
qNRT1.1-F	*Nitrate transporter 1.1*	5′-GGAGGCTCGACTACTTCTAC-3′	XM_037555884
qNRT1.1-R		5′-ATCATGGCCTCCTCGTCAG-3′	
qNRT2.1-F	*High-affinity nitrate transporter 2.1*	5′- CCGGCCAAAAGTTTGCTGAG-3′	XM_037589359
qNRT2.1-R		5′- ATACGTGCTGGGGCGTATTG-3′	
qGS-F	*Glutamine synthetase*	5′- GAGACCGCCGACATCAACAC-3′	XM_037569872
qGS-R		5′- TCATGGAGGTGACGACGTAG-3′	
